# Ferroptosis exacerbates hyperlipidemic acute pancreatitis by enhancing lipid peroxidation and modulating the immune microenvironment

**DOI:** 10.1038/s41420-024-02007-1

**Published:** 2024-05-21

**Authors:** Xinyi Gu, Zhicheng Huang, Xiuzhiye Ying, Xiaodie Liu, Kaiyi Ruan, Sijia Hua, Xiaofeng Zhang, Hangbin Jin, Qiang Liu, Jianfeng Yang

**Affiliations:** 1 The Fourth School of Clinical Medicine, Zhejiang Chinese Medical University, Hangzhou, China; 2 Zhejiang University School of Medicine, Hangzhou, China; 3 Department of Gastroenterology, Affiliated Hangzhou First People’s Hospital, Westlake University School of Medicine, Hangzhou, China; 4 Key Laboratory of Integrated Traditional Chinese and Western Medicine for Biliary and Pancreatic Diseases of Zhejiang Province, Hangzhou, China; 5 Hangzhou Hospital & Institute of Digestive Diseases, Hangzhou, Hangzhou, China

**Keywords:** Immune cell death, Acute pancreatitis, Necroptosis

## Abstract

Abnormal activation of ferroptosis worsens the severity of acute pancreatitis and intensifies the inflammatory response and organ damage, but the detailed underlying mechanisms are unknown. Compared with other types of pancreatitis, hyperlipidemic acute pancreatitis (HLAP) is more likely to progress to necrotizing pancreatitis, possibly due to peripancreatic lipolysis and the production of unsaturated fatty acids. Moreover, high levels of unsaturated fatty acids undergo lipid peroxidation and trigger ferroptosis to further exacerbate inflammation and worsen HLAP. This paper focuses on the malignant development of hyperlipidemic pancreatitis with severe disease combined with the core features of ferroptosis to explore and describe the mechanism of this phenomenon and shows that the activation of lipid peroxidation and the aberrant intracellular release of many inflammatory mediators during ferroptosis are the key processes that regulate the degree of disease development in patients with HLAP. Inhibiting the activation of ferroptosis effectively reduces the intensity of the inflammatory response, thus reducing organ damage in patients and preventing the risk of HLAP exacerbation. Additionally, this paper summarizes the key targets and potential therapeutic agents of ferroptosis associated with HLAP deterioration to provide new ideas for future clinical applications.

## Facts

- The core of ferroptosis is lipid peroxidation of highly expressed unsaturated fatty acids in the cell membrane in response to ferrous ions or lipoxygenase.
- Excessive production of unsaturated fatty acids during HLAP activates ferroptosis via a lipid peroxidation mechanism, which subsequently provides an intrinsic biological environment for the exacerbation of severe pancreatitis.
- Ferroptosis induces the release of inflammatory mediators and DAMPs, which induces a highly proinflammatory state in immune cells and thereby sustains or even exacerbates the inflammatory response.

## Open questions

- Does the membrane of pancreatic acinar cells undergo structural changes during ferroptosis?
- How can molecular targeted drugs related to ferroptosis-mediated inhibition of inflammation be applied to the treatment of HLAP in clinical practice?
- What are the side effects of ferroptosis inhibitors on inhibiting the exacerbation of HLAP?

## Introduction

Acute pancreatitis (AP) is a common gastrointestinal disease caused by factors such as pancreaticobiliary obstruction due to factors such as gallstones, alcohol, and hyperlipidemia. Currently, the global incidence of AP has reached 34/100,000 per year and is increasing, resulting in a heavy medical burden [1]. According to the Atlanta Classification of Acute Pancreatitis, patients can be categorized as having mild acute pancreatitis (MAP), mild severe acute pancreatitis (MSAP), or severe acute pancreatitis (SAP) according to their clinical manifestations and prognosis. MAP is self-limiting and typically resolves spontaneously within 1–2 weeks. However, approximately 20% of MAP cases rapidly become severe with local complications, systemic inflammatory responses and organ failure, and the mortality rate is as high as 20–40% [2]. Compared with other types of AP, hyperlipidemic acute pancreatitis (HLAP) is characterized by a more severe inflammatory response, a high incidence of pancreatic cysts, and prolonged hospitalization. AP-induced alveolar cells continuously release lipase to promote triglyceride esterolysis in adipocytes, resulting in excessive production of unsaturated fatty acids, whereas damaged adipocytes exacerbate alveolar cell necrosis by inhibiting mitochondrial complex I and mitochondrial complex V [3–5]. In addition, the insolubility of triglycerides in the blood and the formation of a microthrombus in the vascular plexus of the pancreas leads to pancreatic infarction, which are key factors in pancreatitis exacerbation [4]. Therefore, early diagnosis and intensive treatment of HLAP are relevant and difficult issues in current research.

Ferroptosis is a new type of regulated cell death that differs from apoptosis, necrosis, and autophagy; requires iron-dependent lipid peroxidation; and is mainly regulated by redox, iron homeostasis and related signaling pathways in a synergistic manner [6]. In recent years, the main mechanisms of ferroptosis have been elucidated. 1. Iron overload and the Fenton reaction generate many free radicals, which mediate oxidative stress, resulting in cellular damage. 2. Lipid peroxidation is induced during lipid metabolism. 3. The depletion of the antioxidant glutathione (GSH) or inactivation of the enzyme glutathione peroxidase 4 (GPX4) promotes lipid peroxidation [7–9]. During the pathogenesis of AP, different types of regulatory cell death (autophagy, necrosis, etc.) have been confirmed to play important roles in disease pathogenesis, leading to differences in patient outcomes [10]. Using ferroptosis inhibitors, Ma and colleagues [11] reduced the degree of inflammation induced by excessive oxidative stress, subsequently alleviating SAP-induced renal injury and suggesting that ferroptosis plays a crucial biological role in AP exacerbation. Although excessive production of unsaturated fatty acids during HLAP activates ferroptosis via a lipid peroxidation mechanism, which in turn provides an intrinsic biological environment for the exacerbation of severe pancreatitis, the specific mechanism has not been determined. Therefore, this article reviews the mechanisms by which ferroptosis interacts with HLAP exacerbation.

## Ferroptosis mediates lipid peroxidation to promote inflammatory responses

The mechanism by which AP progresses to SAP caused by factors such as pancreaticobiliary obstruction, alcohol, and hyperlipidemia consists primarily of untimely activation and destruction of organelles by pancreatic enzymes, excessive release of Ca^2+^-mediated cell death by endoplasmic reticulum stress, and recruitment of immune cells by damaged pancreatic acinar cells to exacerbate inflammation. On this basis, oxidative stress molecules with aberrant lipid accumulation in HLAP are one of the distinct critical mechanisms for exacerbating this type of AP [12]. The major metabolic aberration of HLAP is lipid metabolism, which typically results in a substantial number of plasma high triglyceride-related metabolites, such as triglycerides, free fatty acids, and low-density lipoprotein. Pancreatic lipase can convert triglycerides into free fatty acids. The polyunsaturated fatty acids in free fatty acids can act as substrates for lipid synthesis, causing oxidative stress through the production of reactive oxygen species (ROS) via ester oxygenase. ROS are the core factors of oxidative stress and can act directly on membrane lipids, causing pancreatic cell membrane disintegration, damage to vascular endothelial cells, increased capillary permeability and microcirculation disorders. In addition, ROS can overactivate lymphocytes, resulting in the release of oxygen radicals and an increase in reactive oxidants, further destroying pancreatic alveolar cells, overactivating trypsinogen in alveolar cells, exacerbating pancreatic damage and inducing SAP [13–15]. In recent years, ROS have also been shown to act as signaling molecules, activating proinflammatory genes such as nuclear factor κB (NF-κB) and activated protein-1 (AP-1), amplifying inflammatory responses and exacerbating pancreatic injury [16].

Ferroptosis is thought to result from an imbalance between oxidation and antioxidation, which is characterized by an increase in the iron dependence of ROS, and intracellular ROS oxidation levels exceed the antioxidant levels of GPX4, thereby disrupting redox homeostasis. It is clear that the core of ferroptosis is the lipid peroxidation of highly expressed unsaturated fatty acids in the cell membrane in response to ferrous ions or lipoxygenase, which increases the oxidation level and leads to more lipid peroxidation than antioxidant effects. GPX4, the core enzyme of the antioxidant system, inhibits the antioxidant system, and decreasing the cellular antioxidant capacity by reducing GSH activity and increasing ROS levels in the cell can promote ferroptosis [17–19]. Lipid peroxidation is the main pathway by which ROS are produced during ferroptosis, and the regulation of lipid peroxidation may involve altering lipid peroxidation substrates and lipid oxidation processes. Given that the ROS response is a shared mechanism of AP exacerbation and ferroptosis, we hypothesize that reducing ROS production by using ferroptosis inhibitors would thus affect AP exacerbation, but the detailed underlying mechanisms need to be further explored.

### The accumulation of lipid peroxidation substrates promotes ferroptosis

**Fig. 1:**
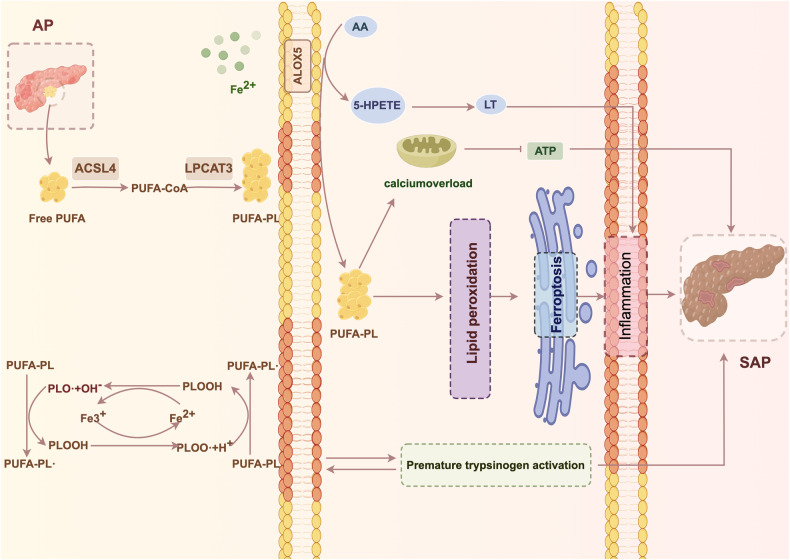
Ferroptosis, lipid peroxidation, and acute pancreatitis. During enzymatic lipid peroxidation, free unsaturated fatty acids are anchored and lengthened at the cell membrane by ACSL4 and LPCAT3, and newly produced long-chain unsaturated fatty acids enter the cytosol to cause calcium overload, which exacerbates pancreatic injury by decreasing ATP production, inducing inflammatory responses, and activating pancreatic enzyme pathways at inappropriate times. In addition, ALOX5 catalyzes the binding of free iron ions to unsaturated fatty acids and the gradual production of inflammatory mediators such as LTs to exacerbate the severity of AP. During nonenzymatic lipid peroxidation, the catabolic products of unsaturated phospholipids use the Fenton reaction to increase cell membrane damage and inappropriately activate pancreatic enzymes to cause SAP (PUFA, polyunsaturated fatty acid; ACSL4, acyl-CoA synthetase long-chain family member 4; LPCAT3, lysophosphatidylcholine acyltransferase 3; AA, arachidonic acid; ALOX5, arachidonate-5-lipoxygenase; 5-HPETE, 5-hydroperoxyeicosatetraenoic acid; LT, leukotriene; PLOO-, phospholipid peroxyl radical; PLOOH, phospholipid hydroperoxide).

Hyperlipidemia is one of the major causes of AP and is significantly associated with disease severity [20, 21]. Excessive accumulation of triglycerides, which are further broken down into fatty acids by lipase, in patients with HLAP induces pancreatic alveolar cell injury by inhibiting mitochondrial function, activating inflammatory responses, and inhibiting autophagic responses [7, 22, 23]. Fatty acids can be categorized as saturated and unsaturated fatty acids. When attached to the surface of the cell membrane, unsaturated fatty acids contribute to lipid peroxidation of the cell membrane, which subsequently leads to the production of lipid ROS levels that exceed antioxidant capacity, thus activating ferroptosis. This process can be categorized into enzymatic and nonenzymatic forms of lipid peroxidation based on the involvement of critical enzymes [24] (Fig. 1).

The main process that occurs in ferroptosis during enzymatic lipid peroxidation is the peroxidation of membrane phospholipids containing unsaturated fatty acids, but free unsaturated fatty acids are not regulated. Anchoring unsaturated fatty acids to the cell membrane is essential for this process, and acyl-CoA synthetase long-chain family member 4 (ACSL4) and lysophosphatidylcholine acyltransferase 3 (LPCAT3) play critical roles [9, 25]. ACSL4 attaches free long-chain unsaturated fatty acids to coenzyme A and then re-esterifies the attached product to phospholipids via LPCAT3, thereby allowing the newly produced long-chain unsaturated fatty acids to enter the cell membrane [6, 26]. In an AP mouse model, exogenous ACSL4-dependent monounsaturated fatty acid supplementation increased the severity of ferroptosis and exacerbated the inflammatory response and organ damage, particularly in the pancreas [27]. High concentrations of unsaturated fatty acids increase intracellular calcium ions in pancreatic alveolar cells, inducing the opening of pore channels in mitochondria, allowing intracytoplasmic components to enter the mitochondria and resulting in the loss of membrane potential, which subsequently hinders the formation of ATP, reduces the discharge of intracytoplasmic calcium ions, and induces calcium overload. Calcium ion overload causes premature activation of pancreatic enzymes, which in turn exacerbates damage to mitochondrial function and aggravates the degree of pancreatic injury, and severe conditions can cause SAP [28–30]. Inhibiting ACSL4 and LPCAT3 expression or reducing the uptake of lipid peroxidation substrates can effectively reduce the occurrence of SAP and alleviate related organ damage caused by an excessive inflammatory response [31].

In addition, the lipoxygenase family is involved in enzymatic lipid peroxidation and plays an important regulatory role in the modulation of ferroptosis. Members of this family are iron-containing dioxygenases that catalyze unsaturated fatty acids and accelerate the binding of oxygen to other polyunsaturated fatty acid compounds, such as arachidonic acid (AA), eicosapentaenoic acid, and docosahexaenoic acid, increasing the extent of lipid peroxidation and thus promoting ferroptosis [32, 33]. There are six different isoforms of human lipoxygenase, of which recombinant arachidonate-5-lipoxygenase (ALOX5) is the most highly regarded given its widespread expression in the circulatory and nervous systems and its role as a key target of ferroptosis [34]. ALOX5 converts AA produced by AP to 5-hydroperoxyeicosatetraenoic acid (5-HPETE), a precursor of the inflammatory signal leukotriene (LT), which progressively induces pancreatic microcirculatory disturbances and inflammatory damage [8, 35]. In SAP, an increase in inflammatory products such as LT, thromboxanes, and prostacyclins, which are metabolites of AA, is responsible for systemic inflammatory response syndrome (SIRS) [21]. Thus, the lipoxygenase family is closely associated with the progression of inflammation, and inhibiting lipoxygenases, particularly ALOX5, may represent a novel anti-inflammatory strategy.

In the nonenzymatic lipid peroxidation pathway, the diallyl hydrogen atom of unsaturated phospholipids is removed, and phospholipid radicals are formed. These radicals then react with molecular oxygen to form phospholipid peroxyl radical (PLOO-). Neighboring unsaturated phospholipids provide hydrogen ions to form phospholipid hydroperoxide (PLOOH) and further stimulate the production of peroxide decomposition products, which destroy adjacent unsaturated phospholipids in the presence of divalent iron, expanding the scope of lipid peroxidation and ultimately disrupting the integrity of the cell membrane [8, 23, 36]. Thus, ferroptosis is more pronounced in organelles with high concentrations of unsaturated fatty acids. During the initiation phase of AP, especially HLAP, pancreatic enzymes, including trypsin, lipase, phospholipase A2 (PLA2), and kinin-releasing enzyme, are inappropriately activated to initiate a cascade reaction in which PLA2 breaks down cell membrane phospholipids and produces lysophosphatidic lecithin and lysophosphatidylcholine. This process causes necrosis in the pancreatic parenchyma and the surrounding adipose tissues, further degradation of triglycerides present in the fats and gradual breakdown of unsaturated fatty acids driven by ferrous ions, thereby generating PLOOH through nonenzymatic lipid peroxidation. PLOOH triggers a new peroxidation chain reaction that exacerbates cell membrane damage and increases the concentrations of pancreatic enzymes to breakdown the pancreatic parenchyma and adipose tissue, constituting a vicious cycle that contributes to the severity of HLAP [37, 38].

In summary, excessive accumulation of lipid substrates increases the amount of unsaturated fatty acids. On the one hand, unsaturated fatty acids use ACSL4 and LPCAT3 to lengthen unsaturated fatty acid chains embedded in the cell membrane or use ALOX5 to accelerate the rate of lipid peroxidation, thereby mediating mitochondrial damage and increasing the release of inflammatory mediators. On the other hand, unsaturated fatty acids have a high affinity for free radicals and can be easily oxidized, resulting in a cascade-like reaction. Therefore, reducing lipid substrates or inhibiting the activities of key enzymes associated with ferroptosis, such as ACSL4, LPCAT3 and ALOX5, may reduce the development of HLAP in patients (Table 1).

**Table 1:** Lipid peroxidation-related targets involved in ferroptosis and their associations with the pathogenesis of acute pancreatitis.

Effect	Molecule	Targets	Acute pancreatitis mechanisms	References
Support ferroptosis	ACSL4	Fatty acids	Calcium overload	[19, 20]
	LPCAT3	Fatty acids	Calcium overload	[6, 25]
	ALOX5	Fatty acids	Inflammation	[8, 33, 34]
	PLOOH	ROS	Membrane damage	[8, 22, 35]
Inhibit ferroptosis	FSP1	CoQ10, NADPH, ESCRT-III	Ca^2+^, Inflammation, Membrane damage	[18, 39, 40]
	NRF2	HO-1, NADPH	Lipid peroxidation	[12, 43, 44]

### Ferroptosis inhibitors attenuate lipid peroxidation

**Fig. 2:**
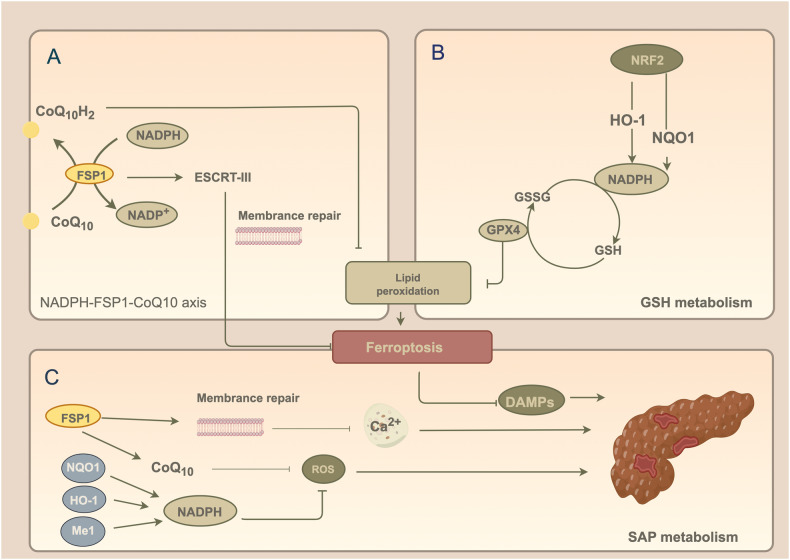
Ferroptosis regulates lipid peroxidation. **A** FSP1 binds to the lipid bilayer and oxidizes NADPH, which increases the conversion of CoQ10 to CoQ10H2 to scavenge ROS, thus inhibiting lipid peroxidation. FSP1 activates ESCRT-III to capture Ca^2+^ and attenuates cytosolic damage in adenohypophysial cells. **B** NRF2 downregulates the severity of ferroptosis through the upregulation of NQO1 and HO-1 and increases NADPH expression. **C** FSP1 and NRF2 can not only alleviate the severity of SAP by inhibiting ferroptosis, but also alleviate pancreatic acinar cell damage by reducing calcium ion overload and ROS levels. (FSP1 fibroblast-specific protein, ESCRT-III endosomal sorting complex required for transport-III, NRF2 nuclear factor erythroid 2-related factor, NQO1 quinone oxidoreductase 1, HO-1 heme oxygenase-1, DAMP damage-associated molecular pattern, GSH glutathione, GPX4 glutathione peroxidase 4).

Doll et al. [39] showed that in addition to the classic ferroptosis pathway, the NADPH-FSP1-CoQ10 signaling pathway specifically regulates ferroptosis in GPX4-knockout mouse embryonic fibroblast Pfa1 cells and human fibrosarcoma HT1080 cells (Table 1). Fibroblast-specific protein 1 (FSP1) is a key ferroptosis inhibitory protein with a canonical myristoylation motif at its N-terminus that can bind to the lipid bilayer and oxidize NADPH, thereby reducing ubiquinone (CoQ10) to dihydroubiquinone to capture lipophilic free radicals for ROS scavenging, thus playing an antioxidant role and inhibiting ferroptosis [19, 39]. CoQ10 is a downstream target of FSP1, and promoting CoQ10 production inhibits ferroptosis. CoQ10 activation in AP mice reduces the levels of inflammatory factors and peroxides in pancreatic tissues, decreases the degree of leukocyte infiltration, and inhibits the expression of extracellular regulated protein kinase (ERK), which mediates the activation of the c-Jun N-terminal kinase (JNK) signaling pathway, thus alleviating the severity of AP [40]. In addition, FSP1 inhibits ferroptosis by activating the membrane repair function of the endosomal sorting complex required for transport-III (ESCRT-III) [41, 42]. ESCRT-III mediates the partial detachment and endocytosis of damaged cell membranes into intracellular or extracellular vesicles, which subsequently reduces calcium ion influx and regulates the imbalance in osmotic pressure inside and outside the membrane to inhibit cell death [43]. As mentioned previously, calcium overload is one of the mechanisms by which AP is converted to SAP. HLAP occurs in response to some degree of cell membrane damage. FSP1 activates ESCRT-III to trap calcium ions and reduce the degree of cell membrane damage, but this mechanism has not yet been fully investigated (Fig. 2).

During oxidative stress, nuclear factor erythroid 2-related factor (NRF2) regulates multiple antioxidant genes involved in cytoprotection [7] (Table 1). NRF2 is activated in unsaturated fatty acid-treated AP mice, resulting in the upregulation of the target genes quinone oxidoreductase 1 (NQO1) and heme oxygenase-1 (HO-1). HO-1 is activated to produce large amounts of ferrous ions, biliverdin and carbon monoxide. Biliverdin is subsequently converted to bilirubin, which inhibits NADPH oxidation. NQO1 reduces NADP^+^ to NADPH, which is a common reductant in organisms and maintains the reduced state of GSH, allowing GPX4 to exert antioxidant effects and reducing the severity of ferroptosis, thereby reducing pancreatic alveolar cell damage and HLAP severity [13, 44, 45]. Therefore, the use of NADPH-targeted drugs and activators or increasing the intake of reducing agents represent promising therapeutic strategies for reducing ferroptosis and SAP (Fig. 2).

### Clinical applications of ferroptosis-mediated lipid peroxidation

Relevant drug studies on the key enzyme regulating ferroptosis have shown that genipin is the product of gardenia glycoside, is hydrolyzed by β-glucosidase, and has anti-inflammatory and proapoptotic effects. In an acute liver injury treatment study, researchers found that after ALOX5 degradation, genipin could reverse the production of metabolites, such as 5-HPETE and AA, thus reducing the levels of lipids involved in ferroptosis and liver inflammation. Peroxidation and the inflammatory response in the liver and silencing of the ALOX5 protein partially eliminated the hepatoprotective effect of genipin, suggesting that genipin reduces the level of lipid peroxidation during ferroptosis by inhibiting ALOX5, which reduces hepatic inflammation and hepatocyte damage [46, 47]. A distinctive feature of Parkinson’s disease is high levels of lipid peroxidation in dopaminergic neurons, and the brain tissue of deceased Parkinson’s patients revealed large areas of ferroptosis, suggesting that ferroptosis is involved in dopaminergic neuron death. Experiments using a Parkinson’s disease model revealed that ferroptosis inducers exacerbated dopaminergic neuron lipoatrophy and that the ACSL4 inhibitor troglitazone (TRO) inhibited lipid peroxidation and restored cell viability [47]. It is clear that these drugs may also attenuate HLAP remission by inhibiting lipid peroxidation-mediated ferroptosis, but this has not been experimentally confirmed in HLAP.

During the regulation of lipid peroxidation, FSP1 has been widely used in experimental studies as a classic inhibitor of ferroptosis, and the antioxidant and anti-inflammatory effects of CoQ10 have been used in clinical practice. NADPH, which is an important cofactor in the reduced GSH-oxidized glutathione (GSSG) cycle, promotes the regeneration of GSH and GPX4 to attenuate lipid peroxidation. New regulatory sites have been identified in ferroptosis studies. NADP^+^-dependent malic enzyme 1 (Me1) binds to NADP^+^ and catalyzes the oxidative decarboxylation of malate to produce NADPH in the presence of divalent ferrous ions to attenuate the severity of ferroptosis; thus, this factor is considered a novel inhibitor of ferroptosis. Fang [48] used this inhibitor in a mouse model of ischemia‒reperfusion-induced liver injury and reported decreases in lipid peroxidation products, the expression of the ferroptosis biomarker Ptgs2, and the concentration of hepatic enzymes, suggesting that Me1 acts as a hepato-protective agent by decreasing the degree of inflammation during ferroptosis. Ding [45] identified metazoan SpoT homolog 1 (MESH1), a homolog of the mammalian genome encoding the bacterial (p)ppGpp hydrolase SpoT and a new target for regulating NADPH, and upregulated MESH1-induced phosphorylation and depletion of NADPH to promote ferroptosis. Therefore, downregulating MESH1 or inducing high NADPH expression is a potential therapeutic strategy for preventing HLAP exacerbation.

## Ferroptosis regulates the immune microenvironment

Like other types of regulatory cell death, ferroptosis destroys the cell membrane through lipid peroxidation, releases damage-associated molecular patterns (DAMPs) and recruits numerous immune cells. High mobility group box-1 (HMGB-1), a DAMP, plays a unique role in ferroptosis. On the one hand, during the early stages of inflammation, cells undergoing ferroptosis release signals that slow the movement of neutrophils in the vasculature and allow them to adhere to the vessel wall, and HMGB-1 recruits other immune cells, such as antigen-presenting cells and macrophages, to activate the inflammatory response [49]. On the other hand, due to elevated levels of ROS during ferroptosis, activation of the MAPK and NF-κB signaling pathways induces macrophages to release HMGB-1 extracellularly, which further increases the inflammatory response by acetylating HMGB-1 in the cytoplasm in an autophagy-dependent manner [25, 50]. Extracellular HMGB-1 can be recognized by the innate immune system during SAP to enhance the inflammatory response and indirectly affect coagulation, and local inflammation damages vascular endothelial cells, causing an imbalance in vascular homeostasis that is characterized by increased vascular permeability, enhanced coagulation, and increased inflammation [51]. DAMPs directly activate platelets and induce platelet aggregation by interacting with neutrophils; in addition, inflammatory signals stimulate endothelial cells to generate more procoagulant factors, resulting in an increase in plasminogen activator inhibitor-1 levels and the expression of coagulation factor V and thrombospondin, which leads to the formation of hypercoagulable states and microcirculatory disturbances [52, 53]. A large amount of HMGB-1 is found in the pancreas in a mouse model of SAP, and inhibiting HMGB-1 with low molecular weight heparin reduces the severity of SAP in mice [54, 55]. Ferroptosis inhibitors also reduce the amount of HMGB-1, further inhibiting leukocyte accumulation and blood hypercoagulation, thereby reducing SAP.

In recent years, ferroptosis has been shown to primarily involve GPX4 to modulate the effects of immune cells on metabolic processes in tumors and inflammatory diseases [49, 56]. GPX4, which is a selenium-rich protein that reduces damage due to oxidative stress by reducing organic peroxides with high molecular weights, is a core ferroptosis protein that is involved in regulating the immune cell microenvironment. The regulation of ferroptosis by inhibiting GPX4 can be mediated by two regulatory mechanisms: the direct inhibition of GPX4 by the classical inhibitor RSL3, which will be described in detail later, and the inhibition of GPX4 by promoting the synthesis of GSH, which consists mainly of glutamate, cysteine, and glycine. Cysteine is not synthesized on its own in organisms and is therefore considered the rate-limiting precursor of GSH. The typical regulatory pathway involves the cystine/glutamate transporter (X_c-_ system), which comprises a heterodimerization protein complex composed of solute carrier family 7 member 11 (SLC7A11) and solute carrier family 3 member 2 (SLC3A2). The X_c-_ system transports one molecule of glutamate out of the cell and one molecule of cystine into the cell and reduces it to cysteine, thus promoting the synthesis of GSH, reducing lipid peroxidation and reducing the expression of ALOX5 to inhibit AA metabolism-related inflammatory factors and reduce the inflammatory response [19, 56].

**Fig. 3:**
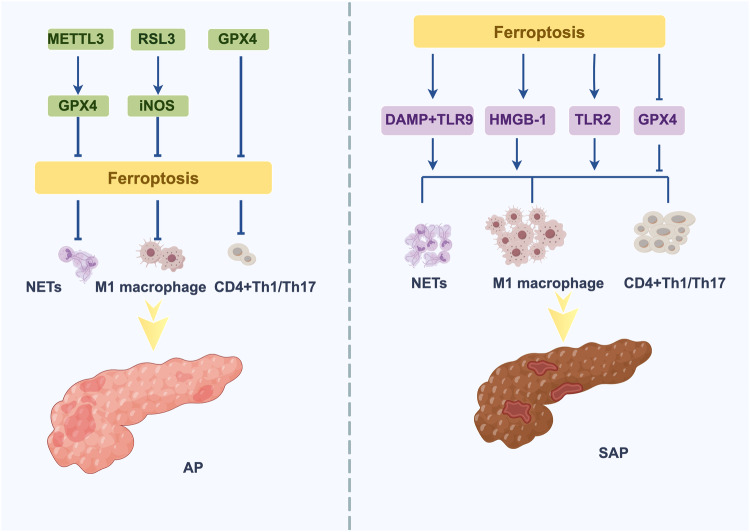
Ferroptosis and SAP-associated inflammatory cells. DAMPs released after ferroptosis bind to TLR4 to increase the production of NETs to exacerbate pancreatic injury. Macrophages use TLR2 to recognize oxidized phospholipids and then phagocytose ferroptotic cells and progressively release HMGB-1, which promotes M1 macrophage polarization to enhance inflammatory responses. GPX4 downregulation promotes immune responses in Th1 and Th17 cells to increase the severity of inflammation. In contrast, low METTL3 expression increases GPX4 expression, which subsequently reduces NET formation by inhibiting ferroptosis. RSL3 alleviates the AP-associated inflammatory response by reducing M1 macrophage polarization through the inhibition of iNOS expression. GPX4 upregulation inhibits the proinflammatory effects of Th1 and Th17 cells (DAMP, damage-associated molecular pattern; TLR4, Toll-like receptor 4; TLR9, Toll-like receptor 9; NETs, neutrophil extracellular traps; HMGB-1, high mobility group box-1; TLR2, Toll-like receptor 2; GPX4, glutathione peroxidase 4; iNOS, inducible nitric oxide synthase; METTL3, methyltransferase-like 3; RSL3, an inhibitor of GPX4).

The inflammatory response plays an important role in the progression of mild to severe HLAP. Multiple signaling pathways are activated within minutes of exposure to proinflammatory signals, such as tumor necrosis factor (TNF) and interleukin (IL), and mediate uncontrolled inflammatory storms that cause damage to the pancreas. Ferroptosis induces the release of inflammatory mediators and DAMPs, which induces a highly proinflammatory state in immune cells and thereby sustains or even exacerbates the inflammatory response. In addition, ferroptosis induces the production of many inflammatory products and enhances inflammation through lipid peroxidation. However, how ferroptosis exacerbates the severity of HLAP remains to be further explored. The mechanism by which ferroptosis exacerbates HLAP severity through different immune cells is shown (Fig. 3).

### Ferroptosis induces M1-like macrophage polarization

Ferrous ions are important for cell proliferation and metabolism and are involved in a variety of cellular functions and differentiation. Macrophages are an important component of intrinsic immunity, and their main function is to phagocytose pathogens to maintain immune homeostasis. Macrophages can be polarized into M1 and M2 macrophages. M1-like macrophages play a proinflammatory role, whereas M2-like macrophages are considered anti-inflammatory cells. It has been shown that M1-like macrophages have a greater ferritin heavy polypeptide/ferritin light polypeptide (FTH/FTL) ratio and lower iron regulatory protein 1 (IRP1) than M2-like macrophages, and it has been hypothesized that iron overload promotes aberrant differentiation in M1-like macrophages [57].

In terms of iron ion uptake, macrophages phagocytose aged erythrocytes and convert them to hemoglobin, and hemoglobin is further converted and releases iron ions. This process increases iron levels in macrophages and tissues, inducing neutrophil migration, activation, and the production of additional inflammatory factors. Among these inflammatory factors, the expression of M1-like macrophage markers, such as interleukin-1β (IL-1β), interleukin-6 (IL-6), and tumor necrosis factor-α (TNF-α), is elevated, and the expression of M2-like macrophage markers, such as transglutaminase 2 (TGM2), is decreased, indirectly suggesting that ferroptosis promotes M1-like macrophage polarization [22, 58]. Macrophages specifically recognize oxidized phospholipids on the surface of ferroptotic cells using the surface membrane receptor Toll-like receptor 2 (TLR2), phagocytose ferroptotic cells and release HMGB-1 [58, 59]. As mentioned previously, this mediator enhances inflammation and induces ROS production by binding to advanced glycosylation end products (AGEs) on macrophages; these macrophages are induced by ROS and ferric ions to stimulate p53 production and enhance p3000/CBP (CREB-binding protein, CBP) acetyltransferase activity, which increases p53 acetylation and polarizes the cells into M1-like macrophages [60]. Ferroptosis also promotes M1-like macrophage polarization by enhancing glycolytic pathways, but this mechanism is still being further explored [58, 61].

M1-like macrophages are core inflammatory cells associated with SAP [62, 63]. Several animal studies have demonstrated that abnormal accumulation of M1-like macrophages exacerbates pancreatic necrosis [64, 65]. RSL3, which is an inhibitor of GPX4, can induce excessive accumulation of M1-like macrophages and exacerbate AP severity by inducing ferroptosis in M2-like macrophages. Kapralow’s study [66] showed that M1-like macrophages exhibit a high level of resistance to RSL3; this phenomenon may be associated with the high expression of the enzyme inducible nitric oxide synthase (iNOS), which is an inhibitor that catalyzes the oxidation of ALOX5 and attenuates ferroptosis, in macrophages. In contrast, M2-like macrophages are more sensitive to RSL3 and exhibit reduced iNOS expression and increased lipid peroxidation, which promotes ferroptosis. Therefore, ferroptosis inhibitors can increase iNOS expression by regulating lipid peroxidation, thereby attenuating the accumulation of iron in M2-like macrophages and moderating ferroptosis to reduce the severity of AP.

### Ferroptosis promotes NET formation

Neutrophils comprise 40–70% of human leukocytes and are the first line of defense against invading pathogens. Neutrophils mediate inflammation using phagocytosis, degranulation, and the formation of neutrophil extracellular traps (NETs). NETs are fibrous meshwork structures composed of cytoplasm, nuclear proteins, various antimicrobial proteins, and inducible factors that can limit pathogen transmission and kill microorganisms. Neutrophil elastase (NE) is closely related to AP-associated inflammation. In the early stages of AP, NE cleaves E-cadherins and increases leukocyte migration, thereby exacerbating the severity of AP. It has been shown that impaired pancreatic microcirculation is positively correlated with the severity of SAP [67]. NETs interact with endothelial cells to achieve microvascular constriction and leukocyte aggregation, further exacerbating pancreatic microcirculation disorders and coagulation abnormalities; in severe cases, microthrombi can form, resulting in exacerbated pancreatic damage and necrosis [68]. In addition, NETs activate trypsinogen mainly through the signal transducer and activator of transcription 3 (STAT3) and matrix metalloproteinase-9 (MMP-9) pathways, increasing the severity of the inflammatory response and reducing the accumulation of neutrophils, which can reduce the severity of AP to a certain extent [69, 70]. Meng [71] attempted to disrupt the skeleton of NETs with deoxyribonuclease I (DNase I) and found that the degree of pancreatic injury, serum amylase levels, and the levels of related inflammatory indicators were significantly reduced in SAP mice.

A correlation between NETs and ferroptosis has recently been demonstrated. Zhang et al. [72] reported that NETs upregulate methyltransferase-like 3 (METTL3), which methylates N6-methyladenosine (m6A) on RNA to regulate gene expression. When METTL3 expression was knocked down, the expression of GPX4, a negative regulator of ferroptosis, was subsequently elevated, suggesting that NETs could promote ferroptosis. Ferroptosis causes DAMPs to be released extracellularly and bind to pattern-recognition receptors. When DAMPs bind to Toll-like receptor 9 (TLR9), the production of NETs is increased, thus constituting a positive cycle. Sulfasalazine is also a powerful agent that mainly induces ferroptosis by inhibiting the Xc- system and ALOX-15. In addition, it can induce the formation of ether esters, leading to the production of more NETs [73]. Moreover, relevant studies have shown that ether esters are key factors in ferroptosis [74]. It is easy to hypothesize that the use of ferroptosis inhibitors to reduce the production of NETs represents a strategy to reverse the shift from AP to SAP, but targeted drugs and clinical studies remain limited and still need to be explored.

### Ferroptosis exacerbates CD4^+^ T-cell functional impairment

T lymphocytes enter the circulatory system after maturation in the thymus and can be divided into cytotoxic T cells, effector T cells, and helper T cells according to their different functions. Among these factors, helper T cells are closely related to the severity of AP. CD4^+^ T cells are activated in pancreatic tissues in early AP. At this stage, there is an increase in the helper T-cell 1 (Th1) marker cytokine interleukin-17 (IL-17), suggesting an increase in Th1 cells, whereas interleukin-4 (IL-4) and interleukin-10 (IL-10) are not induced, suggesting that there are no significant changes in regulatory T cells or helper T-cell 2 (Th2) cells in the splenocytes of mice in the early stage of AP [63]. During the progression of AP, severe necrosis occurs due to the progressive migration of macrophages into pancreatic tissue, and helper T-cell 2 (Th2) cells begin to exert proinflammatory effects and drive T cells to differentiate into Th2 cells, further exacerbating inflammation [75]. Therefore, a severe imbalance in Th1/Th2 cells in tissues and a decrease in CD4^+^ T cells in the peripheral blood can be essential indicators for predicting SAP [76]. Furthermore, helper T-cell 17 (Th17) cells amplify the inflammatory cascade response in AP and initiate SIRS [77]. Xu [78] concluded that during AP, CD4^+^ Th1/Th17 cells induce a proinflammatory response in local pancreatic tissues and progressively amplify the systemic inflammatory response, promoting pancreatic injury.

The effect of ferroptosis on T cells in AP remains to be investigated. However, in antitumor studies, although GPX4 deficiency had no significant effect on T-cell survival in the steady state, it promoted the immune response in helper Th1 cells and helper Th17 cells. In addition, T cells undergoing ferroptosis produce the proinflammatory factor IL-1β, which further promotes the Th17 cell response [79]. GPX4 is a selenium-containing phospholipid peroxidase, and its main function is to reduce peroxidized lipids and thus protect cells. Although GPX4-deficient T cells are able to develop normally in the thymus, the inability of these cells to meet their energy requirements after transferring to the peripheral circulation leads to impaired CD4^+^ T-cell functions [73, 80]. GPX4 is an important factor in ferroptosis, and the use of iron inhibitors to increase GPX4 levels to inhibit the immune response of peripheral CD4^+^ T cells can be used to alleviate SAP.

## Conclusion and outlook

HLAP is more likely to induce microcirculatory disorders and ischemic necrosis in organs. An increase in triglyceride concentration is an independent risk factor for poor prognosis in patients with AP. Triglycerides are broken down by pancreatic lipase into glycerol and unsaturated fatty acids, and high concentrations of unsaturated fatty acids mediate calcium overload, microcirculatory disorders, inflammation, and other mechanisms to exacerbate the degree of pancreatitis injury [81, 82]. Lipid peroxidation is one of the major mechanisms of ferroptosis, and HLAP produces excessive amounts of unsaturated fatty acids to regulate key ferroptosis enzymes to promote oxygen radical production to induce ferroptosis and the release of many inflammatory mediators, which leads to HLAP development. Therefore, HLAP exacerbation is closely related to excessive oxidative stress and inflammatory storms.

The central component of ferroptosis is lipid peroxidation, and the use of extended unsaturated fatty acid chains on cell membranes, activation of lipoxygenase, cascade reactions of unsaturated fatty acids, and inhibition of the NADPH-FSP1-CoQ10 signaling pathway increase intracellular oxidative levels and ROS levels. The accumulation of large amounts of unsaturated fatty acids and an increase in oxidative stress are key factors in HLAP exacerbation, and HLAP patients exhibit large amounts of fatty acids. The catabolism of these fatty acids generates ROS to promote ferroptosis and mediate apoptosis, necrosis, and autophagy. These findings indicate that different regulated forms of cell death can be linked through ROS, providing new clues for us to further explore the mechanism of HLAP exacerbation. In addition, the cytosolic membrane of pancreatic alveolar cells contains a large amount of phospholipids, which increase sensitivity to ferroptosis. However, not all lipid peroxidation causes ferroptosis, and whether the cytosolic membrane undergoes structural changes during ferroptosis and membrane fluidity is reduced needs to be further investigated. Ferroptosis often involves an inflammatory response. On the one hand, oxygen free radicals generated by lipid peroxidation drive inflammatory cell accumulation and activate multiple signaling pathways to exacerbate the inflammatory response. On the other hand, the release of DAMPs from cells undergoing ferroptosis activates the immune system, which leads to a highly proinflammatory state in cells and promotes tissue hypercoagulation, resulting in microcirculatory disturbances that indirectly enhance the inflammatory response. In addition, macrophages, neutrophils, and T cells are important cells involved in the deterioration of HLAP and are common cell populations associated with the inflammatory response caused by ferroptosis. Ferroptosis induces the production of more M1-type macrophages to promote inflammation, and DAMPs recognize and bind to TLR9 to increase NET production, which promotes immune responses and inflammatory storms in helper T1 and T17 cells. Therefore, ferroptosis inhibitors can be used as anti-inflammatory mediators to inhibit inflammation progression by modulating immune factors, immune cells and inflammatory signaling pathways. However, in terms of practical clinical applications, molecularly targeted drugs related to ferroptosis that block inflammation have not been thoroughly investigated, and the beneficial effects of these drugs on HLAP progression, as well as their side effects in vivo, have not been explored.
